# Age-specific genetic and antigenic variations of influenza A viruses in Hong Kong, 2013–2014

**DOI:** 10.1038/srep30260

**Published:** 2016-07-25

**Authors:** Peihua Cao, Chit-Ming Wong, Kwok-Hung Chan, Xiling Wang, King-Pan Chan, Joseph Sriyal Malik Peiris, Leo Lit-Man Poon, Lin Yang

**Affiliations:** 1 Division of Epidemiology and Biostatistics, School of Public Health, The University of Hong Kong, Hong Kong SAR, China; 2 Department of Microbiology, The University of Hong Kong, Hong Kong SAR, China; 3 Department of Biostatistics, School of Public Health and Key Laboratory of Public Health Safety, Fudan University, Shanghai, China; 4 Division of Public Health Laboratory Sciences, School of Public Health, The University of Hong Kong, Hong Kong SAR, China; 5 School of Nursing, The Hong Kong Polytechnic University, Hong Kong SAR, China

## Abstract

Age-specific genetic and antigenic variations of influenza viruses have not been documented in tropical and subtropical regions. We implemented a systematic surveillance program in two tertiary hospitals in Hong Kong Island, to collect 112 A(H1N1)pdm09 and 254 A(H3N2) positive specimens from 2013 to 2014. Of these, 56 and 72 were identified as genetic variants of the WHO recommended vaccine composition strains, respectively. A subset of these genetic variants was selected for hemagglutination-inhibition (HI) tests, but none appeared to be antigenic variants of the vaccine composition strains. We also found that genetic and antigenicity variations were similar across sex and age groups of ≤18 yrs, 18 to 65 yrs, and ≥65 yrs. Our findings suggest that none of the age groups led other age groups in genetic evolution of influenza virus A strains. Future studies from different regions and longer study periods are needed to further investigate the age and sex heterogeneity of influenza viruses.

Influenza viruses undergo frequent antigenic drift, and cause winter epidemics in temperate regions and year-long circulation in tropical and subtropical regions. The World Health Organization (WHO) has established the Global Influenza Surveillance and Response System (GISRS) to track the antigenic change of influenza viruses worldwide, with the aim of guiding the selection of suitable influenza candidate vaccine viruses^1^. In Hong Kong, the influenza surveillance network managed by the Department of Health routinely selected potentially drifted specimens for genetic and antigenic characterization. However, temporal variations of genetic and antigenicity characteristics have never been studied, largely due to the relatively small number of specimens which were subjectively selected based on antigenic drifts and disease severity, rather than from a representative sample of strains currently circulating in the whole population.

Recent large-scale phylogenetic studies have demonstrated co-circulation of different influenza strains in tropical and subtropical regions of Southeast and East Asia, but it remains controversial whether novel viruses first originated from these regions^2^,^3^,^4^. Age discrepancy in susceptibility to different virus subtypes has also been reported^5^. For example, children were found more likely to be infected by A(H1N1)pdm09 as compared to adults, whereas A(H3N2) tended to affect more adults. However, age information has seldom been incorporated into genetic and antigenic surveillance, and to this date few studies have assessed the age difference in terms of genetic and antigenic variations.

In this study, we implemented a systematic surveillance program during 2013–2014, to randomly select a sample of positive specimens of children (aged below 18 years) and adults, from two tertiary hospitals in the Hong Kong Island each week. The systematically collected specimens were expected representative of concurrent circulating strains in the population^6^ and could also allow the investigation of temporal variations of genetic and antigenicity characteristics of concurrently circulating influenza viruses.

## Results

**Figure 1:**
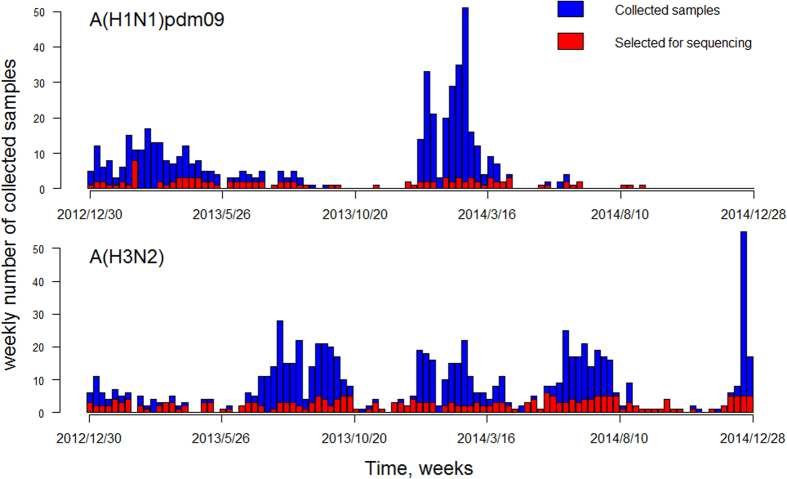
Weekly numbers of collected samples (blue) and those selected for sequencing (red), 2013–2014.

Figure 1 shows the total number of specimens collected by the Queen Mary Hospital microbiology laboratory each week and also the number of specimens selected for sequencing in our study. The demographic characteristics of patients whose specimens were selected for sequencing and Hemagglutinin Inhibition (HI) tests were similar to those unselected patients, except that the percentage of children was slightly higher in the selected than in unselected group for A(H3N2) samples (Table 1). We speculate that this could be due to small numbers of positive specimens in this age group in some weeks.

**Figure 2:**
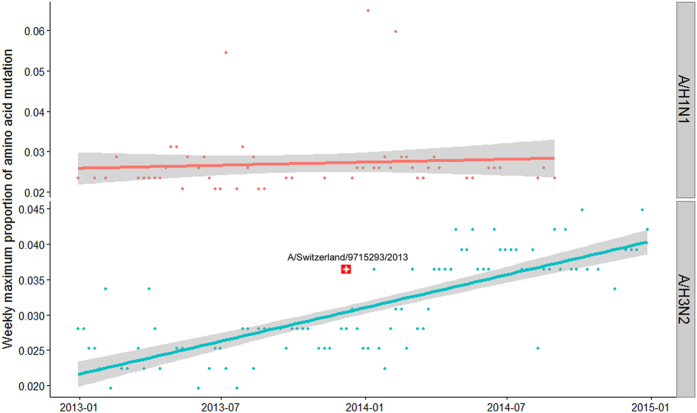
Weekly maximum proportion of amino acid mutations for A(H1N1)pdm09 and A(H3N2), 2013–2014.

Among 366 sequenced strains during 2013–2014, the proportion of amino acid mutations of the HA1 polypeptide for A(H1N1)pdm09 and A(H3N2) was 1.8–6.5% and 1.1–4.5%, respectively. During the study period, the maximum proportion of amino acid mutation per week remained at a relatively low level for subtype A(H1N1)pdm09, but a clear increasing trend was observed for A(H3N2) (Fig. 2).

In the HI tests, over 99% of the isolates had HI titre over 320 for both A(H1N1)pdm09 and A(H3N2), suggesting that no obvious antigenic variants were detected in this study. There were no statistically significant differences in HI titres across age groups of ≤18 yrs, 18 to 65 yrs, and ≥65 yrs [A(H1N1)pdm09 *p* = 0.420; A(H3N2) *p* = 0.798]. No gender difference was found in HI titres [A(H1N1)pdm09 p = 0.304; A(H3N2) p = 0.294] (Table 2). For genetic variations, no statistically significant differences were found across different age or gender groups, in terms of amino acid mutations (Table 2).

Two phylogenetic trees of the HA1 polypeptide sequences from all the A(H1N1)pdm09 and A(H3N2) sequences collected in this study, together with representative strains downloaded from the GISAID EpiFlu™ databank are shown in Supplementary Information. The phylogenetic tree of A(H1N1)pdm09 was separated into eight major genetic clades^7^. All of the A(H1N1)pdm09 strains that were isolated in this study belong to the clade 6 and 7 with a signature substitution of S220T in HA1 polypeptide. Mutations P100S, D114N, S220T, R240Q, and I338V were observed in over 90% of these A(H1N1)pdm09 isolates. Similarly, all the A(H3N2) isolates fell into the clade 3 and 4. There were 7 fixed amino acid mutations (H9Y, Q49R, N161S, Q172H, V202G, Y235S, and N294K) detected in nearly all the A(H3N2) strains (Table 3).

## Discussion

Influenza A viruses are characterized by a high mutation rate^2^. Particularly, HA1 polypeptide exhibited higher sequence variations than HA2, which may be due to selection pressure from the immune system^8^,^9^,^10^. Previous studies had reported that 1% and 0.8% of amino acids in HA1 domain changed per year for H1 and H3, respectively^11^. In our study, although genetic variants were frequently detected, none of these genetic variants were identified as antigenic drift strains from the vaccine composition strains of A/California/07/2009 (H1N1) and A/Victoria/361/2011 (H3N2) recommended during the same season. These findings were consistent with those reported by USCDC that over 90% of A(H1N1)pdm09 and A(H3N2) strains isolated in the 2013–14 season were similar to the vaccine component strains^12^. Interestingly, an antigenic drift strain A/Switzerland/9715293/2013 (H3N2) appeared since January 2015 and caused a relatively severe winter epidemic^13^. Later WHO recommended this new strain as the vaccine composition strain for A(H3N2) in the 2015–2016 season for the Southern Hemisphere^14^. We further compared our sample strains with this new emerging strain and found that some of our sample strains clustered with the A/Switzerland/9715293/2013 (H3N2) into the sub-clade 3C.3 (see phylogenetic tree (b). in Supplementary Information). Our findings of an increasing trend in A(H3N2) mutation rates echo the findings of Shih and colleagues^15^. They also suggested that antigenic change of HA1 appears to be ongoing most of the time with occasional large changes. However, a mutation confers limited antigenic drift and its frequency increases only to a low level in the majority of cases, so that a linear increasing trend of mutations, possibly due to slow accumulation process, may be observed. These findings suggest that the influenza viruses evolve in a gradual process, and mutations at some critical sites that gain the ability of escape from the prior immunity could be crucial for emergence or migration of novel strains into Hong Kong. A future study with a longer study period using our systematic sampling approach could shed more light on whether influenza viruses in Hong Kong evolve through persistent local transmission or repeated introduction from the temperate regions.

Our findings further demonstrated the genetic variability of the influenza A viruses after the pandemic in 2009^16^,^17^. Most A(H1N1)pdm09 isolates obtained during 2013 to 2014 belong to the clade 6 and 7 (see phylogenetic tree (a). in Supplementary Information). This echoes the previous findings of the co-circulation of these clades and high genetic diversity of influenza virus A(H1N1)pdm09 during the 2009–2013 seasons^7^,^18^,^19^. Mutations of P100S, D114N, S202T, S220T, and I338V had frequently been identified in temperate regions including China, France, and Canada, as well as in tropical region of Cuba and Thailand in the same period, whereas R240Q mutation was reported for the first time^18^,^20^,^21^,^22^,^23^. The A(H3N2) strains isolated in Hong Kong during 2013–2014 fell into the A/Victoria/361/2011 genetic clade, and particularly into the clade 3. 97.6% (248/254) of sequences were closely related to the sub-clade 3C and similar to A/Florida/21/2013 strain. The rest (6/254) were grouped into the sub-clade 3B, which were similar to A/Mahajanga/3628/2012 strain (see phylogenetic tree (b). in Supplementary Information). Mutations Q49R, N161S, Q172H, V202G, Y235S, and N294K were also reported in other countries^20^,^21^,^22^,^24^. To our surprise, although genetic changes of influenza viruses were continuously observed, none of them appeared antigenic variants from the vaccine composition strains. This could be due to the relatively short study period and antigenic variants emerging at the end of the period could have been missed. Future studies with intensive collection of sequence data over a longer period, together with more knowledge on the association of genetic and antigenic changes, could make possible the development of a forecast model on newly emerging antigenic variants. Several models have been adopted to predict influenza virus activity, such as the Seasonal Autoregressive Integrated Moving Average (SARIMA) model, Poisson model and SIR model, but most of these only consider environmental factors as the drivers for influenza seasonality^25^,^26^,^27^,^28^,^29^. Our previous study in Hong Kong also demonstrated that the switch of influenza seasonality from one-peak to two-peak patterns coincided with emergence of new A(H3N2) strains, suggesting a link between antigenic change and flu seasonality^30^. Therefore, one future direction of forecast model development could be incorporation of epidemiological and molecular data, similar to those collected in this study, in more sophisticated mathematical models.

Our study also investigated age and gender differences in antigenic and genetic variations. Previous studies demonstrated that the A(H1N1)pdm09 virus was associated with an age shift towards young people^31^,^32^. But inconsistent patterns of gender differences were found across different regions. A study conducted in Australia found more fatal cases were male^33^ while a Canadian study reported that the relative risk of influenza associated deaths in adult women is 1.5 times the risk in men^34^. Some studies observed relatively higher hospitalization risks associated with seasonal influenza A(H3N2) in older men than older women although the difference was not statistically significant^35^,^36^. Boys were found to have a weaker immune response against influenza infections than girls^37^, but this gender difference gradually vanished when they grow into adulthood^38^. In this study we did not find any significant differences across age and gender groups in genetic and antigenic variants of influenza A virus. We have previously analyzed age-specific sentinel surveillance data of laboratory confirmed influenza in Hong Kong, and found highly synchronized influenza seasonal epidemics across age groups in terms of the seasonal patterns, epidemic timing and durations^5^. Previous studies proposed that influenza seasonal variations could be driven by seasonal change of environmental factors (temperature, humidity, etc.), host immunity and antigenic change of viruses^25^,^39^. Hence this study provides more evidence to support the age synchrony in antigenic evolution of influenza viruses, which could partially explain the findings of our previous ecological study. Furthermore, our study has exemplified the plausibility of collecting individual demographical data during laboratory surveillance, and a better understanding could be achieved on age and gender synchrony of influenza infections when such data become available in a wider range.

There are several limitations in our study. First, the majority of samples were taken from inpatients admitted into two public hospitals. However, there is no strong evidence to suggest that influenza A viruses circulating in the community are different from those isolated from inpatients^40^,^41^. Second, we only sequenced the HA1 polypeptide of HA due to the limited budget. However, HA2 polypeptide of HA, neuraminidase (NA) and other proteins could also affect the genetic and antigenic characteristics of influenza A viruses, which have not been explored in this study. Third, HI tests are often criticized for its low sensitivity as compared to other tests such as micro-neutralization tests, which might lead to relatively low efficiency in detecting antigenic variants. Fourth, there are other available measurements for antigenic distance, such as antigenic maps which requires pairwise HI tests across different reference strains. However, given the high titres in HI tests, it is unlikely to observe obvious differences between these newly isolated strains if multiple reference strains are used. When potential antigenic variants appear in future studies, more sensitive tests and additional reference strains should be considered.

In conclusion, by using an age-stratified random selection strategy, we detected some genetic variants in Hong Kong, but no obvious antigenic variants from the WHO vaccine strains during the study period of 2013 to 2014. Further studies are warranted to integrate both antigenic profiles of locally circulating strains and other factors, allowing us to gain a better understanding on the mechanism of seasonal influenza epidemics.

## Materials and Methods

### Sample collection

**Figure 3:**
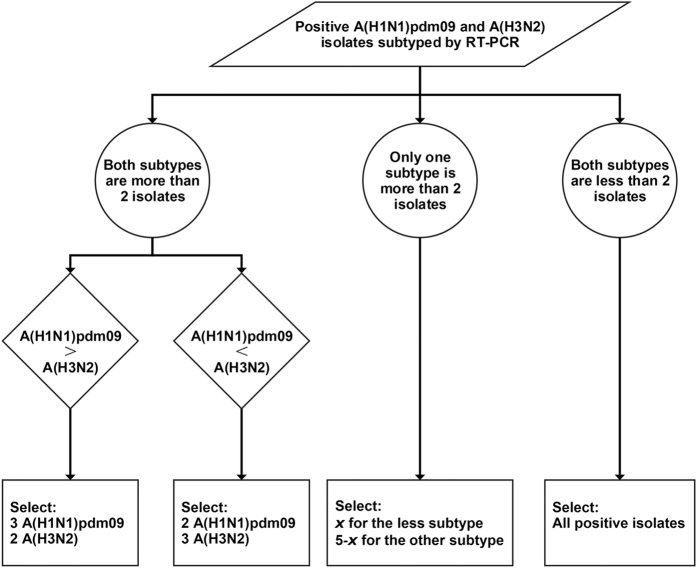
Flow chart of sample selection in this study.

The microbiology laboratory of Queen Mary Hospital routinely collects the nasopharyngeal aspirates (NPA) or nasopharyngeal swabs (NPS) from patients who are admitted into two tertiary hospitals (Queen Mary Hospital and Pamela Youde Nethersole Eastern Hospital) in Hong Kong Island, with influenza like symptoms (temperature >38 °C, cough and/or sore throat). These two public hospitals accept approximately 80% of all hospitalizations in Hong Kong Island which has a total population of one million^42^. Both NPA and NPS specimens were divided into two aliquots for direct immunofluorescence (IF) test and RT-PCR respectively. Direct IF D3 Ultra 8 DFA respiratory virus screening and identification kit (Diagnostic Hybrid, OH) was used to detect antigens of the specimens, and then viewed at a magnification of 400 under epifluorescent illumination of an Eurostar III plus (EUROIMMUN AG, Lübeck, Germany) fluorescence microscope^43^. Influenza A positive specimens were subsequently sub-typed into A(H3N2) and A(H1N1)pdm09 by RT-PCR^44^. Each week up to five influenza A positive specimens were selected for genetic sequencing between January 2013 to December 2014. If there were more than two positive isolates for each of two subtypes A(H1N1)pdm09 or A(H3N2), two were randomly selected from the subtype with less total number of positive specimens and three from the other. If one or two positive specimens were found for one subtype in that week, all of them were selected, and the rest were from the other subtype to get a total of five specimens. If there were five or less positive specimens in one week, all of them were selected. This selection procedure is shown in Fig. 3. In order to assess the age difference, positive specimens were randomly selected from different age groups of ≤18 yrs, 18 to 65 yrs, and ≥65 yrs, if any. For example, if there were three specimens to be selected from A(H3N2) positive specimens from three age groups, one would be randomly selected from each age group. If there were only two age groups, then one extra sample would be selected from the age group with more positive specimens. If there were five or less positive specimens in one week, all of them would be selected. Because in some weeks there were few or zero positive specimens isolated in some age groups, age distribution might be different between selected and unselected specimens due to the high chance of being selected in these age groups.

The selected A(H1N1)pdm09 and A(H3N2) positive specimens were then isolated by cell culture on MDCK cells^45^, and subsequently sequenced using the Big Dye Terminator v3.1 Cycle Sequencing Kit. Only HA1 polypeptide was sequenced because mutations frequently occur in this polypeptide. It contains 329 amino acids, of which131 are located at or close to five antibody epitopes and are the critical sites determining the virus antigenicity^10^,^46^. Bioedit version 7.2.5 was used to assemble and edit sequences (http://www.mbio.ncsu.edu/bioedit/bioedit.html). We chose the representative isolates which had mutations identified from genetic sequencing, for further hemagglutinin-inhibition (HI) tests. The reference strains used in the HI tests were A/California/07/2009 (H1N1) and A/Victoria/361/2011 (H3N2), which were WHO recommended vaccine composition strains in the 2012/13 and 2013/14 seasons for the Northern Hemisphere. The HI tests started with 1:10 dilution of antiserum and the HI titre less than 40 was regarded as the threshold to define the antigenic variants^47^. Phylogenetic trees were produced by the *G T R* + *G* + *Γ* model of amino acid substitutions incorporated in the MrBayes v.3.2.5 software and visualized with the FigTree v.1.4.2^48^. These gene sequences were compared with a sample of gene sequences that had been globally isolated in recent years, from the GISAID EpiFlu database (http://www.platform.gisaid.org) (Table S1). These reference strains selected from the database were representative of the major genetic clades defined by WHO^7^.

During the study period of January 1^st^, 2013 to December 31^st^, 2014, a total of 2,115 patients admitted to the two public hospitals in Hong Kong Island presented with influenza like symptoms (temperature >38 °C, cough and/or sore throat). Of these, 491 were confirmed infected with A(H1N1)pdm09 and 803 with A(H3N2). We randomly selected 366 positive specimens for sequencing [112 A(H1N1)pdm09 positive and 254A(H3N2) positive]. 56 of A(H1N1)pdm09 and 72 of A(H3N2) sequenced specimens with potential meaningful point mutations were further selected for HI test.

### Statistical analysis

Genetic variation was measured by the proportion of amino acid mutations of the HA1 polypeptide relative to the reference strains, for A(H1N1)pdm09 and A(H3N2) subtypes, respectively^21^. The proportion of amino acid mutations was defined as


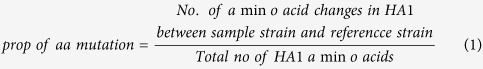


Antigenic variation was assessed by the HI tests against the reference strains. The Fisher exact tests were used to compare the rates across age and gender groups. In this study statistical significance was defined as *p* < 0.05. Statistical analyses were performed using R software version 3.0.0^49^.

Ethical approval for this study was obtained from the Institutional Review Board of the University of Hong Kong/Hospital Authority Hong Kong West Cluster (Reference Number: UW 11-290). Informed consent was obtained from all subjects. All experiments were carried out in accordance with the approved guidelines.

## Additional Information

**How to cite this article**: Cao, P. *et al*. Age-specific genetic and antigenic variations of influenza A viruses in Hong Kong, 2013–2014. *Sci. Rep.* **6**, 30260; doi: 10.1038/srep30260 (2016).

## Supplementary Material

Supplementary Information

## Figures and Tables

**Table 1:** Comparison of demographic characteristics of selected and unselected samples by season.

Sub-type (Year)	Characteristic	Selected case	Unselected case	*p*-value*
		*number*(%)		
A(H1N1)pdm09				
2013	Total specimens	67 (100%)	172 (100%)	
	Age (years)			0.86
	≤18	36 (49%)	98 (57%)	
	19–64	20 (23%)	50 (29%)	
	≥65	11 (28%)	24 (14%)	
	Male	35 (52%)	83 (48%)	0.68
2014	No. of patients	45 (100%)	207 (100%)	
	Age (years)			0.14
	≤18	22 (49%)	73 (35%)	
	19–64	15 (33%)	71 (34%)	
	≥65	8 (18%)	63 (30%)	
	Male	23 (51%)	105 (51%)	0.91
A(H3N2)				
2013	Total specimens	116 (100%)	240 (100%)	
	Age (years)			0.001
	≤18	53 (46%)	67 (28%)	
	19–64	23 (20%)	45 (19%)	
	≥65	40 (34%)	128 (53%)	
	Male	65 (56%)	116 (48%)	0.21
2014	No. of patients	138 (100%)	309 (100%)	
	Age (years)			<0.001
	≤18	56 (41%)	65 (21%)	
	19–64	28 (20%)	55 (18%)	
	≥65	54 (39%)	189 (61%)	
	Male	73 (53%)	154 (50%)	0.62

^*^Pearson’s Chi-square tests.

**Table 2:** Comparison of haemagglutinin inhibition test results and HA1 amino acid mutations of A(H1N1)pdm09 and A(H3N2) samples across age and gender, against the reference strains A/California/07/2009 (H1N1) and A/Victoria/361/2011 (H3N2).

Subtype	No. of samples	Dilution of antiserum in HI tests				*p*-value*	Genetic variations (Amino acid mutations)	*p*-value#
		160	320	640	≥1280		*Mean*±*SD*	
H1N1	Age (years)					0.42		0.265
	≤18	0	1	11	13		9.34 ± 1.79	
	19–64	0	1	12	8		10.49 ± 3.55	
	≥65	0	1	7	2		9.63 ± 1.01	
	Male	0	0	15	10	0.304	9.50 ± 2.23	0.276
	Female	0	3	15	13		10.00 ± 2.62	
H3N2	Age (years)					0.798		0.136
	≤18	0	8	14	7		10.11 ± 2.59	
	19–64	1	5	7	4		9.79 ± 2.57	
	≥65	0	7	10	9		10.66 ± 2.48	
	Male	0	14	15	12	0.294	10.13 ± 2.54	0.451
	Female	1	6	16	8		10.38 ± 2.59	

^*^Fisher exact tests.

^#^One-way ANOVA.

**Table 3:** Common amino acid substitutions observed in antigenic sites of HA1 polypeptide of A(H1N1)pdm09 and A(H3N2) influenza virus isolates, Hong Kong, 2013–2014.

Subtype	Antigenic site	Original amino acid substitution	New amino acid substitution	Mutated strains *number (percent)*
H1N1	100	P	S	111 (99.11)
	114	D	N	103 (91.96)
	202	S	T	103 (91.96)
	220	S	T	112 (100)
	240	R	Q	111 (99.11)
	338	I	V	110 (98.21)
H3N2	9	H	Y	250 (98.43)
	49	Q	R	249 (98.03)
	161	N	S	247 (97.24)
	172	Q	H	248 (97.64)
	202	V	G	253 (99.61)
	235	Y	S	252 (99.21)
	294	N	K	252 (99.21)
