# Supplementary material for: Age-specific genetic and antigenic variations of influenza A viruses in Hong Kong, 2013–2014
Source: Sci Rep. 2016 Jul 25;6:30260. doi: 10.1038/srep30260 (PMC4958999; doi:10.1038/srep30260)
Supplement: Supplementary Information [file srep30260-s1.pdf]

Supplementary information

Title: Age-specific genetic and antigenic variations of influenza A virusesin Hong Kong, 2013-2014

Peihua Cao, Chit-Ming Wong, Kwok-Hung Chan, Xiling Wang, King-Pan Chan, Joseph Sriyal Malik Peiris, Leo Lit-Man Poon, Lin Yang

Phylogenetic trees

(a). Phylogenetic tree based on influenza A(H1N1)pdm09 viruses HA1 polypeptide sequences. 112 strains from the present study were denoted by blue (for Hong Kong 2013 strains) colour and black (for Hong Kong 2014 strains) colour. 20 strains selected from GISAID EpiFlu database and 2 vaccine strains (A/California/07/2009 and A/New York/20/2009) were denoted by red colour. Phylogenetic tree was produced by the $GTR+G+\Gamma$ model of amino acid substitutions incorporated in the MrBayes v.3.2.5 software. Generation number and sampling frequency were set to 200,000 and 5,000, respectively.

(b). Phylogenetic tree based on influenza A(H3N2) viruses HA1 polypeptide sequences. 254 strains from the present study were denoted by blue (for Hong Kong 2013 strains) colour and black (for Hong Kong 2014 strains) colour. 26 strains selected from GISAID EpiFlu database and 2 vaccine strains (A/Victoria/361/2011 and A/Switzerland/97152931/2013) were denoted by red colour. Phylogenetic tree was produced by the $GTR+G+\Gamma$ model of amino acid substitutions incorporated in the MrBayes v.3.2.5 software. Generation number and sampling frequency were set to 200,000 and 5,000, respectively.

Table S1. List of selected strains from the GISAID EpiFlu database.

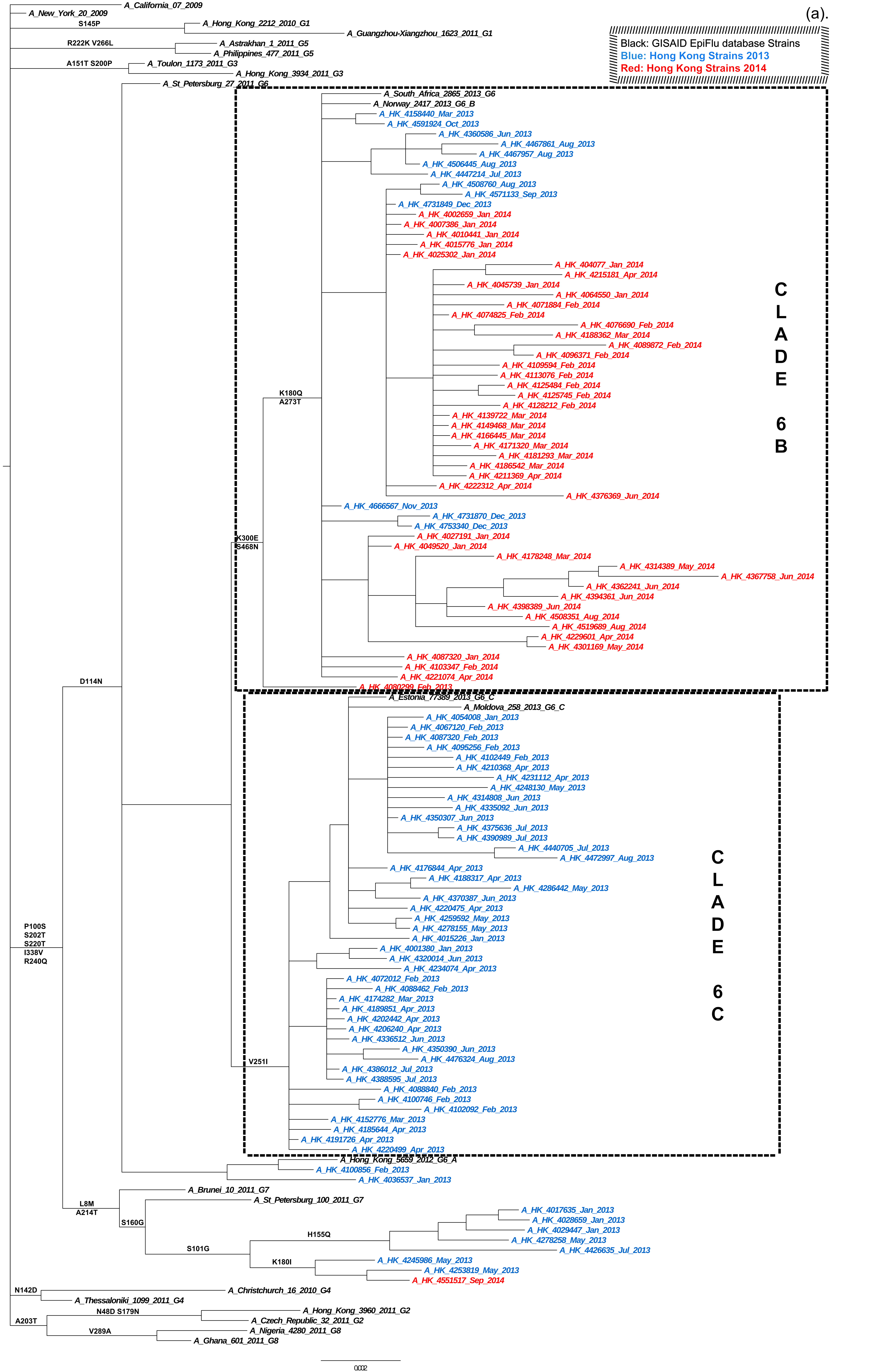
(a).
Black: GISAID EpiFlu database Strains
Blue: Hong Kong Strains 2013
Red: Hong Kong Strains 2014
A_California_07_2009
A_New_York_20_2009
S145P
A_Hong_Kong_2212_2010_G1
A_Guangzhou-Xiangzhou_1623_2011_G1
R222K V266L
A_Astrakhan_1_2011_G5
A_Philippines_477_2011_G5
A151T S200P
A_Toulon_1173_2011_G3
A_Hong_Kong_3934_2011_G3
A_St_Petersburg_27_2011_G6
A_South_Africa_2865_2013_G6
A_Norway_2417_2013_G6_B
A_HK_4158440_Mar_2013
A_HK_4591924_Oct_2013
A_HK_4360586_Jun_2013
A_HK_4467861_Aug_2013
A_HK_4467957_Aug_2013
A_HK_4506445_Aug_2013
A_HK_4447214_Jul_2013
A_HK_4508760_Aug_2013
A_HK_4571133_Sep_2013
A_HK_4731849_Dec_2013
A_HK_4002659_Jan_2014
A_HK_4007386_Jan_2014
A_HK_4010441_Jan_2014
A_HK_4015776_Jan_2014
A_HK_4025302_Jan_2014
A_HK_404077_Jan_2014
A_HK_4215181_Apr_2014
A_HK_4045739_Jan_2014
A_HK_4064550_Jan_2014
A_HK_4071884_Feb_2014
A_HK_4074825_Feb_2014
A_HK_4076690_Feb_2014
A_HK_4188362_Mar_2014
A_HK_4089872_Feb_2014
A_HK_4096371_Feb_2014
A_HK_4109594_Feb_2014
A_HK_4113076_Feb_2014
A_HK_4125484_Feb_2014
A_HK_4125745_Feb_2014
A_HK_4128212_Feb_2014
A_HK_4139722_Mar_2014
A_HK_4149468_Mar_2014
A_HK_4166445_Mar_2014
A_HK_4171320_Mar_2014
A_HK_4181293_Mar_2014
A_HK_4186542_Mar_2014
A_HK_4211369_Apr_2014
A_HK_4222312_Apr_2014
A_HK_4376369_Jun_2014
K180Q
A273T
C
L
A
D
E
6
B
A_HK_4666567_Nov_2013
A_HK_4731870_Dec_2013
A_HK_4753340_Dec_2013
K300E
S468N
A_HK_4027191_Jan_2014
A_HK_4049520_Jan_2014
A_HK_4178248_Mar_2014
A_HK_4314389_May_2014
A_HK_4367758_Jun_2014
A_HK_4362241_Jun_2014
A_HK_4394361_Jun_2014
A_HK_4398389_Jun_2014
A_HK_4508351_Aug_2014
A_HK_4519689_Aug_2014
A_HK_4229601_Apr_2014
A_HK_4301169_May_2014
A_HK_4087320_Jan_2014
A_HK_4103347_Feb_2014
A_HK_4221074_Apr_2014
A_HK_4080299_Feb_2013
D114N
A_Estonia_77389_2013_G6_C
A_Moldova_258_2013_G6_C
A_HK_4054008_Jan_2013
A_HK_4067120_Feb_2013
A_HK_4087320_Feb_2013
A_HK_4095256_Feb_2013
A_HK_4102449_Feb_2013
A_HK_4210368_Apr_2013
A_HK_4231112_Apr_2013
A_HK_4248130_May_2013
A_HK_4314808_Jun_2013
A_HK_4335092_Jun_2013
A_HK_4350307_Jun_2013
A_HK_4375636_Jul_2013
A_HK_4390989_Jul_2013
A_HK_4440705_Jul_2013
A_HK_4472997_Aug_2013
A_HK_4176844_Apr_2013
A_HK_4188317_Apr_2013
A_HK_4286442_May_2013
A_HK_4370387_Jun_2013
A_HK_4220475_Apr_2013
A_HK_4259592_May_2013
A_HK_4278155_May_2013
A_HK_4015226_Jan_2013
A_HK_4001380_Jan_2013
A_HK_4320014_Jun_2013
A_HK_4234074_Apr_2013
C
L
A
D
E
6
C
P100S
S202T
S220T
I338V
R240Q
A_HK_4072012_Feb_2013
A_HK_4088462_Feb_2013
A_HK_4174282_Mar_2013
A_HK_4189851_Apr_2013
A_HK_4202442_Apr_2013
A_HK_4206240_Apr_2013
A_HK_4336512_Jun_2013
A_HK_4350390_Jun_2013
A_HK_4476324_Aug_2013
V251I
A_HK_4386012_Jul_2013
A_HK_4388595_Jul_2013
A_HK_4088840_Feb_2013
A_HK_4100746_Feb_2013
A_HK_4102092_Feb_2013
A_HK_4152776_Mar_2013
A_HK_4185644_Apr_2013
A_HK_4191726_Apr_2013
A_HK_4220499_Apr_2013
A_Hong_Kong_5659_2012_G6_A
A_HK_4100856_Feb_2013
A_HK_4036537_Jan_2013
A_Brunei_10_2011_G7
A_St_Petersburg_100_2011_G7
L8M
A214T
S160G
A_HK_4017635_Jan_2013
A_HK_4028659_Jan_2013
A_HK_4029447_Jan_2013
H155Q
A_HK_4278258_May_2013
A_HK_4426635_Jul_2013
S101G
A_HK_4245986_May_2013
K180I
A_HK_4253819_May_2013
A_HK_4551517_Sep_2014
N142D
A_Christchurch_16_2010_G4
A_Thessaloniki_1099_2011_G4
N48D S179N
A_Hong_Kong_3960_2011_G2
A203T
A_Czech_Republic_32_2011_G2
V289A
A_Nigeria_4280_2011_G8
A_Ghana_601_2011_G8
0.002

(b).

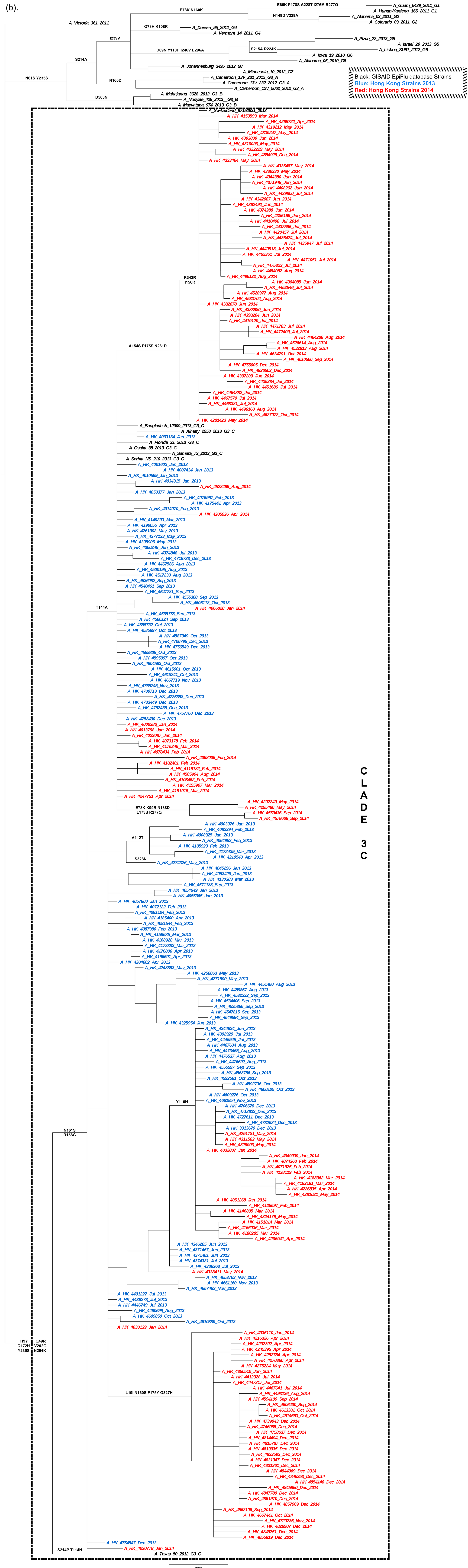
Black: GISAID EpiFlu database Strains
Blue: Hong Kong Strains 2013
Red: Hong Kong Strains 2014
E78K N160K
E66K P178S A228T I276M R277Q
N149D V229A
Q73H K108R
I239V
S214A
D69N Y110H I246V E296A
S215A R224K
N61S Y235S
N160D
D503N
K342R
I156R
A154S F175S N261D
T144A
E78K K99R N138D
L173S R277Q
A112T
S328N
Y110H
N161S
R158G
H9Y
Q172H
Y235S
Q49R
V202G
N294K
L19I N160S F175Y Q327H
S214P T114N
CLADE 3C
0.002

Table S1. List of selected strains from GISAID EpiFlu database.

	Clade	Strains	Clade	Strains	Clade	Strains
A(H1N1)pdm09	1	A/Hong Kong/2212/2010<br>A/Guangzhou-Xiangzhou/1623/2011	4	A/Christchurch/16/2010<br>A/Thessaloniki/1099/2011	6 C	A/Estonia/77389/2013<br>A/Moldova/258/2013
	2	A/Hong Kong/3960/2011<br>A/Czech Republic/32/2011	5	A/Philippines/477/2011<br>A/Astrakhan/1/2011	7	A/St-Petersburg/100/2011<br>A/Brunei/10/2011
	3	A/Hong Kong/3934/2011<br>A/Toulon/1173/2011	6	A/St-Petersburg/27/2011<br>A/South Africa/2865/2013		
			6 A<br>6 B	A/Hong Kong/5659/2012<br>A/Norway/2417/2013	8	A/Nigeria/4280/2011<br>A/Ghana/601/2011
A(H3N2)	1	A/Guam/6439/2011<br>A/Hunan-Yanfeng/165/2011	3 B	A/Mahajanga/3628/2012<br>A/Maevatanana/974/2013<br>A/Athens/GR/112/2012<br>A/Nosy Be/429/2013/	4	A/Darwin/95/2011<br>A/Vermont/14/2011
	2	A/Alabama/03/2011<br>A/Colorado/03/2011			5	A/Plzen/22/2013<br>A/Israel/20/2013<br>A/Alabama/05/2010
	3 A	A/Cameroon/13V-231/2012<br>A/Cameroon/13V-232/2012<br>A/Cameroon/12V-5062/2012	3 C	A/Bangladesh/12009/2013<br>A/Almaty/2958/2013<br>A/Florida/21/2013<br>A/Osaka/38/2013<br>A/Samara/73/2013<br>A/Serbia/NS-210/2013	6	A/Lisboa/SU91/2012<br>A/Iowa/19/2010
					7	A/Johannesburg/3495/2012<br>A/Minnesota/10/2012
